# Comparative Effects of Live and Heat-Killed *Lacticaseibacillus paracasei* HP7 on Intestinal Motility, Barrier-Related Markers, and Gut Microbiota in Delayed Transit Mice

**DOI:** 10.4014/jmb.2605.05041

**Published:** 2026-07-01

**Authors:** Hyeonjun Gwon, Hyeonji Kim, Joo-Yun Kim, Jae-Jung Shim, Jae-Hwan Lee

**Affiliations:** R&BD Center, hy Co., Ltd., 22, Giheungdanji-ro 24beon-gil, Giheung-gu, Yongin-si 17086, Gyeonggi-do, Korea

**Keywords:** *Lacticaseibacillus paracasei* HP7, Postbiotics, Intestinal motility, Loperamide-induced delayed transit, Gut microbiota, Predicted functional profiling

## Abstract

This study evaluated the effects of live and heat-killed *Lacticaseibacillus paracasei* HP7 on intestinal motility, host physiological responses, and gut microbiota in a mouse model of delayed intestinal transit. BALB/c mice were treated with live HP7 (HP7L), heat-killed HP7 (HP7K), or bisacodyl following loperamide administration. HP7L and HP7K improved intestinal motility-related parameters, including fecal output, stool consistency, fecal moisture content, and charcoal meal transit. HP7 treatment also modulated circulating gastrointestinal hormones and inflammatory cytokines, with changes in serotonin, ghrelin, peptide YY, IL-6, and IL-17A. In intestinal tissues, HP7 regulated the expression of genes related to neuroendocrine signaling, water transport, mucus production, and tight junction integrity, including *Ghrl*, *Tac1*, *Slc6a4*, *Aqp3*, *Muc2*, *Tjp1*, *Tjp2*, and *Ocln*. Histological analysis further showed reduced colonic tissue alterations and improved structural parameters. Microbiota analysis revealed that HP7L exerted broader effects on microbial community structure, whereas HP7K induced more selective taxonomic changes. Predicted functional profiling suggested that both HP7L and HP7K were associated with shifts in carbohydrate metabolism-related pathways, with HP7K showing additional predicted enrichment of pathways related to redox-associated metabolism, lipid metabolism, and microbial substrate utilization. These findings suggest that heat-killed HP7 retains biological activity and has potential as a postbiotic candidate for regulating intestinal motility.

## Introduction

Efficient gastrointestinal (GI) motility is a fundamental physiological process coordinated by the enteric nervous system, intestinal smooth muscle, and neuroendocrine signaling [1, 2]. Appropriate intestinal transit supports nutrient absorption, microbial fermentation, and fluid balance, whereas impaired motility prolongs luminal retention and increases water reabsorption [3, 4]. These changes can disturb microbial metabolism, expose the intestinal mucosa to prolonged luminal stress, and contribute to impaired bowel movement and disruption of intestinal physiological homeostasis [5]. Because intestinal transit is closely linked to mucosal barrier integrity, immune regulation, and microbial activity, strategies that restore motility while preserving mucosal and microbial homeostasis have received increasing attention [6-8].

Probiotics, particularly lactic acid bacteria, have been widely studied for their ability to support gastrointestinal function and microbial balance [9, 10]. Several mechanisms have been proposed, including regulation of serotonin signaling, stimulation of short-chain fatty acid production, modulation of intestinal barrier function, and interaction with the resident gut microbiota [11]. Although probiotics have shown beneficial effects, their application can be influenced by microbial viability, processing conditions, storage stability, and host-related safety considerations [12, 13]. These practical issues have encouraged interest in postbiotics, which are preparations of inanimate microorganisms or their components that confer health benefits to the host [14]. Compared with live probiotics, postbiotics may offer advantages in stability, safety, and standardization. In addition, non-viable microbial preparations can interact with intestinal epithelial and immune cells, modulate pattern recognition receptor-mediated signaling, and regulate barrier and inflammatory responses without requiring colonization [15].

*Lacticaseibacillus paracasei* HP7 has previously been reported to improve gastric dysfunction and delayed gastric emptying in experimental models [16, 17]. These previous studies primarily addressed upper gastrointestinal dysfunction and functional dyspepsia-related parameters, including gastric emptying and digestive factor regulation. However, whether live and heat-killed HP7 exert comparable or distinct effects on lower intestinal transit, colonic barrier-related responses, and gut microbial community structure has not been clarified. [17]. This distinction is important because lower gastrointestinal motility is not determined solely by transit speed but is also associated with fecal water content, colonic tissue structure, mucus barrier status, inflammatory tone, gastrointestinal hormone signaling, and microbial metabolic potential [18, 19]. Therefore, an integrated evaluation is required to determine whether HP7 affects delayed intestinal transit through coordinated regulation of host physiological and microbiota-associated responses.

To address these gaps, the present study investigated the comparative effects of live and heat-killed *L. paracasei* HP7 in a loperamide-induced delayed intestinal transit mouse model [20, 21]. In addition to intestinal transit parameters, colonic histological alterations, mucosal barrier-related factors, inflammatory cytokines, gastrointestinal hormones, gut microbiota composition, and predicted microbial functional profiles were comprehensively analyzed. Through integrated physiological and microbiome analyses, this study aimed to characterize the effects of HP7 on delayed intestinal transit and to evaluate the potential of heat-killed HP7 as a postbiotic candidate for lower gastrointestinal function.

## Materials and Methods

### Preparation of Live HP7 and Heat-Killed HP7

The probiotic strain, *L. paracasei* HP7, was cultivated in de Man, Rogosa, and Sharpe (MRS) medium (Difco Laboratories, USA) at 37°C for 24 h. After incubation, the bacterial cells were harvested by centrifugation at 3,000 × g for 20 min at 4°C. The cell pellet was washed twice with sterile phosphate-buffered saline (PBS) and resuspended in sterile PBS. The live HP7 suspension was prepared fresh daily immediately prior to each oral administration. To prepare heat-killed HP7, the bacterial suspension was subjected to thermal inactivation in a water bath at 100°C for 20 min with continuous agitation. Before heat inactivation, viable bacterial counts were determined by the serial dilution and plate count method, and the dose of HP7K was calculated based on the pre-inactivation viable cell count. The complete loss of viability was confirmed by plating the heat-treated suspension on MRS agar, followed by incubation at 37°C for 48 h. After heat inactivation, HP7K was aliquoted, stored at -20°C, and used within 7 days after preparation. Before administration, each aliquot was thawed at 4°C and gently mixed to ensure a homogeneous suspension. Repeated freeze-thaw cycles were avoided by using single-use aliquots.

### Animal Study Design

Male BALB/c mice (6 weeks old) were purchased from Dooyeol Biotech (Republic of Korea). The animals were acclimated for 1 week under controlled conditions (temperature 20 ± 2°C, humidity 50 ± 10%, 12-h light/dark cycle), with standard diet and water provided ad libitum. After acclimation, mice were randomly divided into five groups (*n* = 8 per group). To induce delayed intestinal transit, loperamide was orally administered at 5 mg/kg per dose twice daily for 7 days, except in the normal control (NOR) group. Loperamide administration was discontinued after the 7-day induction period, and mice then received PBS, bisacodyl, HP7L, or HP7K for 14 consecutive days. The experimental groups consisted of a NOR group receiving PBS without loperamide, a loperamide-treated control group (CON) receiving PBS, a positive control group treated with bisacodyl (10 mg/kg/day; BSC), and two treatment groups receiving either live *L. paracasei* HP7 (HP7L) or heat-killed *L. paracasei* HP7 (HP7K). HP7L was administered once daily by oral gavage at a dose of 1.0 × 10^9^ CFU/kg/day. HP7K was administered at 1.0 × 10^9^ CFU-equivalent/kg/day, calculated based on the viable cell count before heat inactivation. At the end of the experiment, mice were euthanized by CO_2_ inhalation, and biological samples were collected immediately thereafter. All animal procedures were approved by the Institutional Animal Care and Use Committee (IACUC) of hy Co. Ltd. (Approval No. ACE-2026-0001-Y).

### Fecal Analysis and Stool Consistency Scoring

To evaluate bowel function and intestinal transit, fecal samples were collected at two experimental stages. The first collection was performed after 7 days of loperamide administration to confirm successful induction of delayed intestinal transit. The second collection was conducted after the 14-day intervention period. For quantification of fecal output, fecal pellets were collected individually from each mouse over a 24-h period with free access to food and water, and the total number of fecal pellets was counted and expressed as the number of pellets per mouse. Stool consistency was evaluated using a previously described fecal scoring method based on pellet morphology and hardness, with minor modifications [22]. Fecal pellets were graded using five-point scale as follows: 1, normal and well-formed pellets with a smooth surface; 2, slightly dry but well-formed pellets; 3, moderately dry pellets with visible surface cracks; 4, hard and dry pellets with an irregular shape; and 5, extremely dry, small, and hard pellets. Following collection, fecal moisture content was measured using a Halogen Moisture Analyzer (HX204; Mettler Toledo, Switzerland). Fresh fecal samples were evenly placed on the sample pan and dried at 105°C using the standard drying program until the automatic switch off criterion was reached.

### Charcoal Meal Test

To evaluate gastrointestinal motility, a charcoal meal transit assay was performed. The marker meal consisted of 5% (w/v) activated charcoal suspended in 10% (w/v) gum arabic solution (Sigma-Aldrich, USA). Following the final treatment administration, mice were orally administered 0.2 mL of the charcoal suspension by gavage. After 30 min, the entire small intestine from the pylorus to the cecum was carefully excised. The distance traveled by the charcoal marker and the total length of the small intestine were measured. The intestinal transit ratio (%) was calculated using the following formula: Intestinal transit ratio (%) = distance traveled by the charcoal marker / total length of the small intestine × 100.

### Serum Hormone and Cytokine Analysis

At sacrifice, blood samples were collected via cardiac puncture and allowed to clot at room temperature for 30 min. Serum was obtained by centrifugation at 3,000 × g for 15 min at 4°C and stored at -80°C until analysis. Serum serotonin (5-hydroxytryptamine, 5-HT) levels were quantified using a 5-HT ELISA kit (MyBioSource, USA). Serum levels of ghrelin, peptide YY (PYY), interleukin-1β (IL-1β), tumor necrosis factor-α (TNF-α), interleukin-6 (IL-6), and interleukin-17A (IL-17A) were measured using a Luminex multiplex assay (Luminex platform; R&D Systems, USA). All assays were performed according to the manufacturer’s instructions.

### Intestinal Gene Expression Analysis by RT-qPCR

To investigate region-specific intestinal functions, gene expression analysis was performed separately in the small intestine and colon tissues. Total RNA was extracted using the easy-spin™ Total RNA Extraction Kit (iNtRON Biotechnology, Republic of Korea) according to the manufacturer’s instructions, and RNA concentration and purity were determined by spectrophotometric analysis. Complementary DNA (cDNA) was synthesized from 1 μg of total RNA using the Maxime™ RT PreMix Kit (iNtRON Biotechnology). qRT-PCR was carried out using TaqMan™ Gene Expression Assays (Thermo Fisher Scientific, USA) on a QuantStudio real-time PCR system (Thermo Fisher Scientific). Relative gene expression levels were calculated using the 2^-ΔΔCt^ method, and gene expression was normalized to *Gapdh* (glyceraldehyde-3-phosphate dehydrogenase, Mm99999915_g1) as an endogenous control. *Gapdh* was used as the endogenous control because its Ct values remained stable across the experimental groups under the present experimental conditions. The following TaqMan assay IDs were used: *Ghrl* (ghrelin; Mm00612524_m1), *Tac1* (tachykinin 1; Mm01166996_m1), *Slc6a4* (serotonin transporter; Mm00439391_m1), *Aqp3* (aquaporin 3; Mm01208559_m1), and *Muc2* (mucin 2; Mm01276676_m1). In addition, tight junction–related genes were analyzed using *Tjp1* (tight junction protein 1; Mm00493699_m1), *Tjp2* (tight junction protein 2; Mm00495699_m1), and *Ocln* (occludin; Mm00500912_m1). Colon tissues were analyzed for the expression of *Aqp3*, *Muc2*, *Tjp1*, *Tjp2*, and *Ocln*, whereas small intestine tissues were analyzed for *Ghrl*, *Tac1*, and *Slc6a4*.

### Histological Analysis and Scoring

For histological evaluation, distal colon tissues were fixed in 10% neutral buffered formalin (Sigma-Aldrich), embedded in paraffin, sectioned, and stained with hematoxylin and eosin (H&E). Tissue processing, H&E staining, slide scanning, and histopathological assessment were performed by DooYeol Biotech. Histopathological assessment was performed in a blinded manner with respect to group allocation. Representative images and morphometric measurement fields were selected from well-oriented, intact mucosal regions without tissue folding, sectioning artifacts, or staining artifacts. Histological alterations were assessed based on four parameters: muscle integrity, mucosal barrier integrity, goblet cell density, and submucosal inflammation. Each parameter was scored on a four-point scale as follows: 0, no apparent alteration; 1, mild alteration; 2, moderate alteration; and 3, severe alteration. The scores for the four parameters were summed to obtain the total histological score for each sample. Smooth muscle layer thickness and crypt length were subsequently measured from the scanned H&E images using ImageJ software (version 1.53t) as morphometric indicators of distal colonic structural changes.

### Gut Microbiota Analysis by 16S rRNA Gene Sequencing

Microbial genomic DNA was extracted from fecal contents and subjected to 16S rRNA gene sequencing targeting the V3–V4 hypervariable regions using the MiSeq i100 system (Illumina, USA). Sequencing was performed by Macrogen Inc. (Republic of Korea) using 300 bp paired-end reads. Library preparation, sequencing, and initial quality control were performed by Macrogen Inc. Raw sequencing reads were processed using the QIIME 2 pipeline (version 2023.9) in a Linux-based environment. Demultiplexed sequences were quality-filtered and denoised using the DADA2 plugin to generate amplicon sequence variants (ASVs). For DADA2 processing, the first 5 bases were trimmed from both forward and reverse reads, and forward and reverse reads were truncated at 280 bp and 250 bp, respectively, based on the sequence quality profiles. After DADA2 denoising and chimera removal, the number of non-chimeric reads ranged from 7,268 to 17,636 reads per sample. Alpha and beta diversity analyses were performed using a rarefied ASV table at a sampling depth of 7,200 reads per sample, which was selected based on the minimum retained read depth. Representative sequences were aligned using MAFFT, and a phylogenetic tree was constructed with FastTree for downstream diversity analyses. Taxonomic assignment was performed using a pretrained Naive Bayes classifier trained on the SILVA 138 99% OTU reference sequences. Fecal microbiota analysis was performed using endpoint fecal samples collected after the 14-day intervention period. Baseline fecal microbiota profiling before loperamide induction or treatment was not performed. Alpha diversity metrics were calculated to assess within-sample microbial diversity, and statistical differences among groups were evaluated using the Kruskal-Wallis test. Beta diversity was assessed using weighted and unweighted UniFrac distance metrics and visualized by principal coordinates analysis (PCoA), with group differences evaluated using permutational multivariate analysis of variance (PERMANOVA). Differentially abundant taxa among experimental groups were identified using Linear Discriminant Analysis Effect Size (LEfSe). Associations between the relative abundances of gut microbial taxa and host biochemical parameters were assessed using Spearman’s rank correlation analysis in R software (version 3.6.6). The raw 16S rRNA gene sequencing data have been deposited in the NCBI Sequence Read Archive under BioProject accession number PRJNA1479854.

### Predictive Functional Profiling of Gut Microbiota

Predictive functional profiling of the microbial communities was performed using the QIIME 2 plugin *q2-picrust2* (version 2023.9), which implements the PICRUSt2 (Phylogenetic Investigation of Communities by Reconstruction of Unobserved States) pipeline. Predicted metagenomes were normalized by 16S rRNA gene copy number and annotated against the Kyoto Encyclopedia of Genes and Genomes (KEGG) Orthology and MetaCyc metabolic pathway databases. Differentially enriched predicted functional features were identified using Linear Discriminant Analysis Effect Size (LEfSe). Features with *p* < 0.05 and a Linear Discriminant Analysis (LDA) score > 2.0 were considered significant. For each database, significant KEGG Orthology and MetaCyc pathway features were ranked by LDA score, and the top 10 features with the highest LDA scores were visualized.

### Statistical Analysis

All data are presented as the mean ± standard deviation (SD). Statistical analyses were performed using GraphPad Prism software (version 6; GraphPad Software, USA). Comparisons between two groups were performed using an unpaired Student’s t-test. Comparisons among three or more groups were analyzed by one-way analysis of variance (ANOVA) followed by Tukey’s multiple comparison test. Alpha diversity was analyzed using the Kruskal-Wallis test, and beta diversity was assessed using PERMANOVA based on UniFrac distances. Differentially abundant taxa and predicted functional features were identified using LEfSe. Spearman’s rank correlation analysis was used to assess associations between microbial taxa and host physiological parameters. A *p* value < 0.05 was considered statistically significant. Different superscript letters indicate significant differences among groups at *p* < 0.05.

## Results

### Induction of Impaired Intestinal Motility and Effects of HP7 on Fecal Parameters

**Fig. 1:**
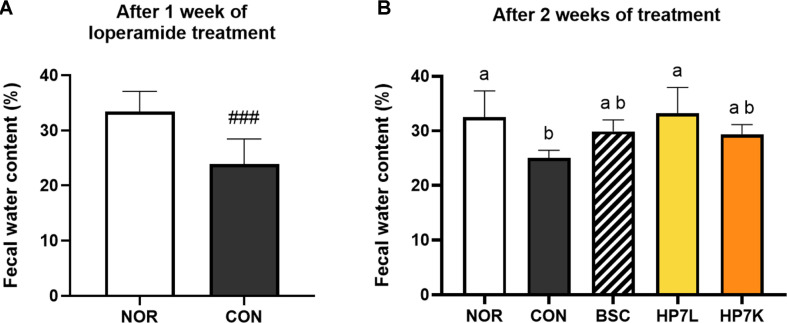
Changes in fecal water content during loperamide induction and HP7 administration. (**A**) Fecal water content measured after 1 week of loperamide administration to confirm the induction of delayed intestinal transit. (**B**) Fecal water content measured after the 14-day intervention period in mice treated with BSC, HP7L, or HP7K. Data are presented as mean ± SD. For panel A, statistical significance between the NOR and CON groups was determined using an unpaired Student’s t-test, and ### *p* < 0.001 compared with the NOR group. For panel B, different letters indicate significant differences among groups according to one-way ANOVA followed by Tukey’s multiple comparison test (*p* < 0.05).

Loperamide administration (5 mg/kg, per dose twice daily for 7 days) significantly reduced fecal moisture content from 33.37 ± 3.70% in the NOR group to 23.87 ± 4.60% in the CON group consistent with the induction of a loperamide-induced delayed-transit phenotype (Fig. 1A). After the 14-day intervention, fecal moisture in the CON group remained significantly lower than that in the NOR group (25.50 ± 1.10% compared with 33.82 ± 5.09%). HP7L treatment restored fecal moisture to a level comparable to that of the NOR group (33.23 ± 1.94%), HP7K and BSC showed numerical increases in fecal moisture content compared with the CON group, but these differences did not reach statistical significance (Fig. 1B).

**Fig. 2:**
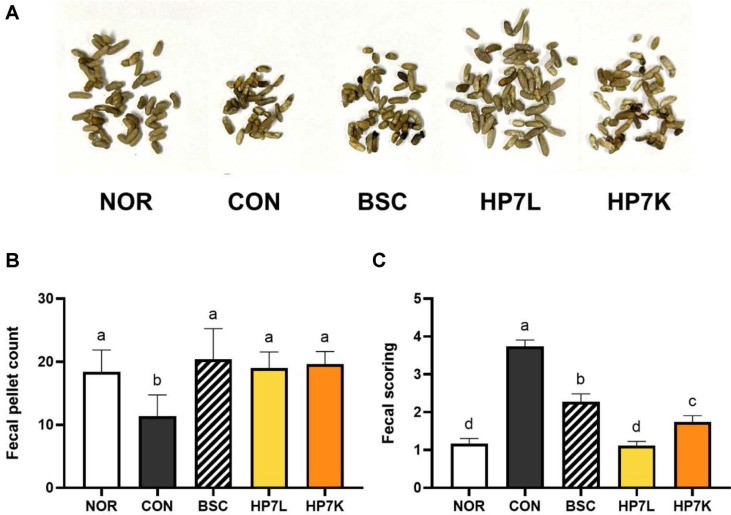
Effects of HP7L and HP7K on fecal pellet output and stool consistency. (**A**) Representative photographs of fecal pellets collected over a 24-h period from each experimental group. (**B**) Total number of fecal pellets excreted per mouse over 24 h. (**C**) Fecal consistency score based on pellet morphology and hardness. Data are presented as mean ± SD. Different letters indicate significant differences among groups according to one-way ANOVA followed by Tukey’s multiple comparison test (*p* < 0.05).

Loperamide also significantly affected fecal pellet output and stool consistency (Fig. 2A). The CON group showed a marked reduction in 24-h pellet counts and increased fecal scores compared with the NOR group, consistent with reduced fecal output and altered stool consistency. Both HP7L and HP7K increased fecal output to levels comparable to the NOR group (Fig. 2B). Stool consistency was significantly improved by HP7L, while HP7K and BSC showed numerical improvements (Fig. 2C).

### Enhancement of Small Intestinal Motility in the Charcoal Meal Assay

**Fig. 3:**
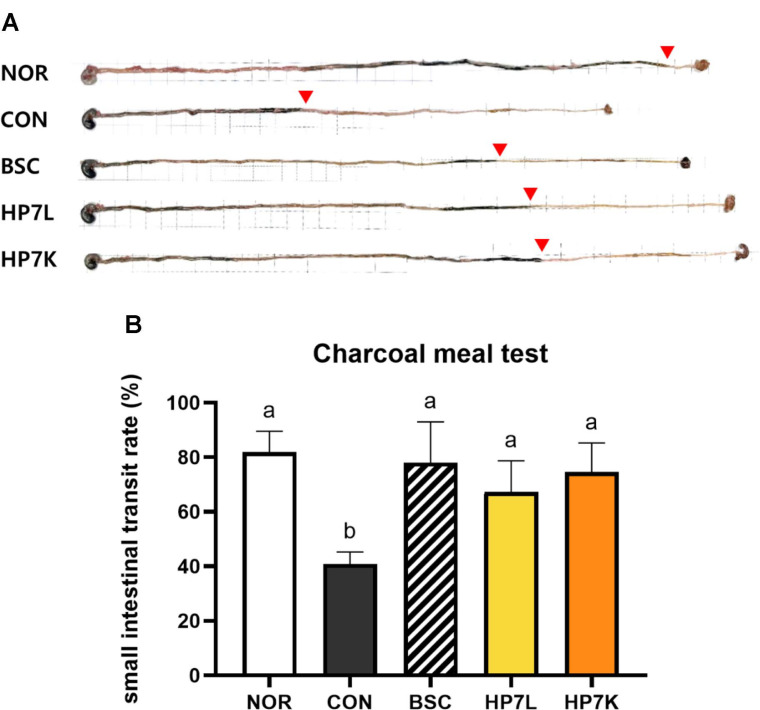
Effects of HP7L and HP7K on small intestinal transit in the charcoal meal assay. (**A**) Representative images showing charcoal meal progression through the small intestine. Red arrowheads indicate the leading edge of the charcoal marker. (**B**) Small intestinal transit rate calculated as the distance traveled by the charcoal marker relative to the total length of the small intestine. Data are presented as mean ± SD. Different letters indicate significant differences among groups according to one-way ANOVA followed by Tukey’s multiple comparison test (*p* < 0.05).

The charcoal meal transit assay showed that loperamide administration markedly delayed small intestinal transit (Fig. 3A). The intestinal transit rate was significantly lower in the CON group than in the NOR group, decreasing from 81.99 ± 7.53% to 40.85 ± 4.49% (Fig. 3B). Treatment with BSC increased the transit rate to 78.01 ± 14.91%, indicating recovery of loperamide-induced delayed transit. HP7 administration also significantly increased charcoal progression compared with the CON group, with transit rates of 67.31 ± 11.38% in the HP7L group and 73.32 ± 8.59% in the HP7K group. The HP7K group showed a numerically higher transit rate than the HP7L group.

### HP7 Modulates Circulating Gastrointestinal Hormones and Inflammatory Cytokines

**Fig. 4:**
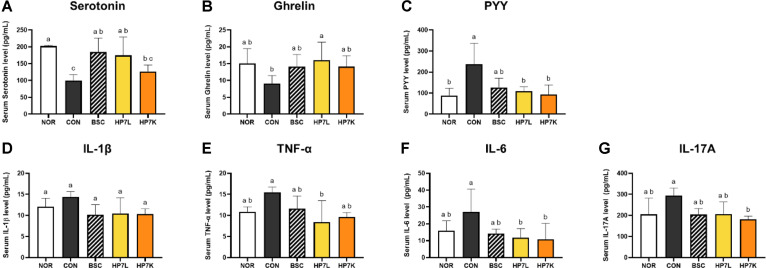
Effects of HP7L and HP7K on serum gastrointestinal hormones and inflammatory cytokines. Serum concentrations of (**A**) serotonin, (**B**) ghrelin, (**C**) peptide YY (PYY), (**D**) IL-1β, (**E**) TNF-α, (**F**) IL-6, and (**G**) IL-17A were measured in each experimental group. Data are presented as mean ± SD. Different letters indicate significant differences among groups according to one-way ANOVA followed by Tukey’s multiple comparison test (*p* < 0.05).

To evaluate the effects of HP7 on circulating biomarkers associated with intestinal motility and inflammation, serum levels of serotonin, ghrelin, PYY, and inflammatory cytokines were quantified (Fig. 4A–4G).

Loperamide treatment significantly decreased serum serotonin levels from 202.1 ± 2.25 pg/mL in the NOR group to 100.14 ± 17.56 pg/mL in the CON group. Administration of BSC and HP7L significantly increased serotonin levels compared with the CON group, whereas HP7K showed a non-significant increasing trend (Fig. 4A). Similarly, serum ghrelin levels were significantly increased by HP7L treatment compared with the CON group (Fig. 4B). In contrast, peptide YY (PYY), an inhibitory regulator of gastrointestinal transit, was markedly increased following loperamide administration [23]. This increase was significantly attenuated in both the HP7L and HP7K groups, while the BSC group showed a decreasing tendency (Fig. 4C).

Serum inflammatory cytokine levels tended to be higher in the CON group than in the NOR group. Serum IL-1β levels did not differ significantly among the groups, and the treatment groups showed only minor numerical changes compared with the CON group (Fig. 4D). Serum TNF-α levels were significantly reduced by HP7L treatment compared with the CON group, whereas the BSC and HP7K groups showed non-significant decreasing trends (Fig. 4E). Serum IL-6 levels were significantly reduced by both HP7L and HP7K treatments compared with the CON group (Fig. 4F). Serum IL-17A levels were significantly reduced by HP7K treatment compared with the CON group, while the BSC and HP7L groups showed non-significant decreasing trends (Fig. 4G).

### HP7 Modulates Gene Expression Related to Intestinal Motility in the Small Intestine

**Fig. 5:**
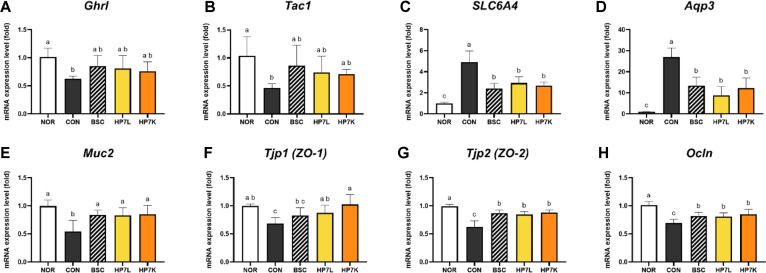
Effects of HP7L and HP7K on intestinal motility-related, water transport-related, and barrier-related gene expression. Relative mRNA expression levels of small intestinal motility-related genes, including (**A**) *Ghrl*, (**B**) *Tac1*, and (**C**) *Slc6a4*, and colonic water transport- and barrier-related genes, including (**D**) *Aqp3*, (**E**) *Muc2*, (**F**) *Tjp1* (ZO-1), (**G**) *Tjp2* (ZO-2), and (**H**) *Ocln*, were determined by RT-qPCR. Gene expression was normalized to *Gapdh* and expressed as fold change relative to the NOR group. Data are presented as mean ± SD. Different letters indicate significant differences among groups according to one-way ANOVA followed by Tukey’s multiple comparison test (*p* < 0.05).

To investigate molecular changes associated with the motility regulatory effects of HP7, mRNA levels of motility related genes were analyzed in small intestinal tissues (Fig. 5A–5C). Loperamide administration significantly downregulated *Ghrl* and *Tac1*, while markedly upregulating *Slc6a4* compared with the NOR group. In the CON group, *Ghrl* and *Tac1* levels decreased to 0.63 ± 0.05-fold and 0.46 ± 0.07-fold, respectively, suggesting impaired peristaltic signaling. HP7L and HP7K increased *Ghrl* levels to 0.81 ± 0.23-fold and 0.76 ± 0.17-fold respectively, and *Tac1* levels to 0.74 ± 0.29-fold and 0.71 ± 0.09-fold, respectively. In contrast, *Slc6a4* expression was elevated in the CON group to 4.91 ± 1.08-fold and was reduced to 2.94 ± 0.57-fold and 2.70 ± 0.31-fold following HP7L and HP7K treatment, respectively.

### HP7 Regulates Water Transport and Barrier-Related Gene Expression in the Colon

To further evaluate the effects of HP7 on colonic water transport and barrier function, the mRNA levels of water transport- and barrier-related genes were analyzed in colon tissues (Fig. 5D–5H). *Aqp3* expression was markedly increased in the CON group to 26.91 ± 4.44-fold compared with 1.03 ± 0.14-fold in the NOR group, showing a marked increase in *Aqp3* expression following loperamide administration. HP7L and HP7K significantly reduced *Aqp3* expression to 8.72 ± 4.16-fold and 12.22 ± 4.81-fold, respectively. In contrast, *Muc2*, *Tjp1*, *Tjp2*, and *Ocln* expression levels were decreased in the CON group compared with the NOR group. HP7L and HP7K increased the expression of these barrier-related genes compared with the CON group. Notably, *Tjp1* expression in the HP7K group reached 1.03 ± 0.18-fold, which was comparable to the NOR grou*p* value of 1.00 ± 0.04-fold.

### HP7 Attenuates Loperamide-Induced Histological Alterations in the Distal Colon

**Fig. 6:**
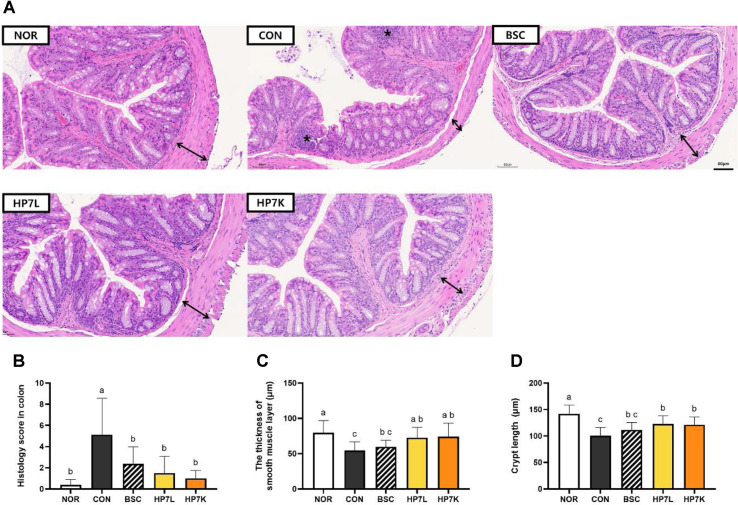
Effects of HP7L and HP7K on histological alterations in the distal colon. (**A**) Representative hematoxylin and eosin (H&E)-stained images of distal colon tissues from each experimental group. Asterisks indicate inflammatory cell infiltration, and double-headed arrows indicate smooth muscle layer thickness. Scale bar = 60 μm. (**B**) Total histological score calculated based on muscle integrity, mucosal barrier integrity, goblet cell density, and submucosal inflammation. (**C**) Smooth muscle layer thickness. (**D**) Crypt length. Data are presented as mean ± SD. Different letters indicate significant differences among groups according to one-way ANOVA followed by Tukey’s multiple comparison test (*p* < 0.05).

Histological examinations of distal colon tissues showed mucosal alterations and inflammatory cell infiltration in the CON group compared with the NOR group (Fig. 6A). The total histological score was higher in the CON group than in the NOR group, with values of 5.13 ± 3.44 and 0.38 ± 0.52, respectively (Fig. 6B). HP7L and HP7K treatment reduced the histological score compared with the CON group. Morphometric analysis showed that loperamide administration decreased smooth muscle layer thickness and crypt length compared with the NOR group. HP7L and HP7K treatment increased both parameters compared with the CON group (Fig. 6C and 6D).

### HP7 Modulates Gut Microbial Diversity and Community Structure

**Fig. 7:**
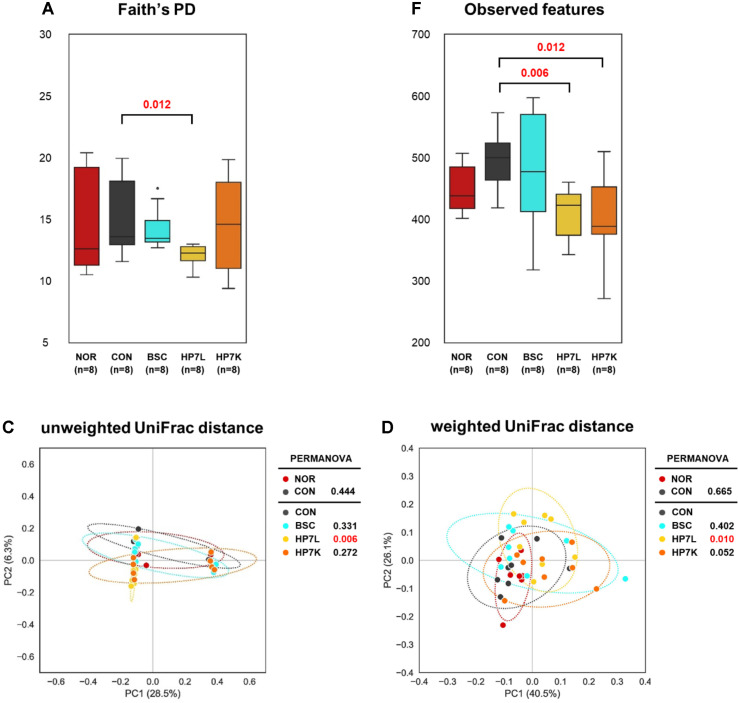
Effects of HP7L and HP7K on gut microbial alpha and beta diversity. Alpha diversity was assessed using (**A**) Faith’s phylogenetic diversity (Faith’s PD) and (**B**) observed features. Beta diversity was evaluated using principal coordinates analysis (PCoA) based on (**C**) unweighted UniFrac distance and (**D**) weighted UniFrac distance. Statistical differences in alpha diversity were evaluated using the Kruskal-Wallis test. Differences in beta diversity among groups were assessed using PERMANOVA. The *p* values shown in the panels indicate the statistical significance of pairwise comparisons.

The effects of HP7 on the fecal microbiota were assessed by analyzing α- and β-diversity indices (Fig. 7A-7D). For α-diversity, Faith’s PD and observed features showed numerical increases in the CON group compared with the NOR group, although these differences were not statistically significant. HP7L treatment significantly decreased both Faith’s PD and observed features compared with the CON group (*p* < 0.05). HP7K treatment significantly decreased observed features (*p* < 0.05), while Faith’s PD showed a numerical decrease compared with the CON group (Fig. 7A and 7B). Beta diversity analysis showed changes in microbial community structure among the experimental groups (Fig. 7C and 7D). In the unweighted UniFrac distance analysis, no significant separation was observed between the NOR and CON groups, whereas HP7L treatment resulted in a significant difference compared with the CON group according to PERMANOVA (*p* < 0.01). In the weighted UniFrac distance analysis, no significant difference was observed between the NOR and CON groups, although partial separation was observed in the PCoA plot. HP7L treatment significantly altered the weighted UniFrac profile compared with the CON group (*p* < 0.05), whereas the BSC group showed no significant difference. HP7K showed a trend toward a difference in weighted UniFrac distance compared with the CON group (*p* = 0.052).

### HP7 Modulates Taxonomic Profiles and Microbial Biomarkers in Fecal Microbiota

**Fig. 8:**
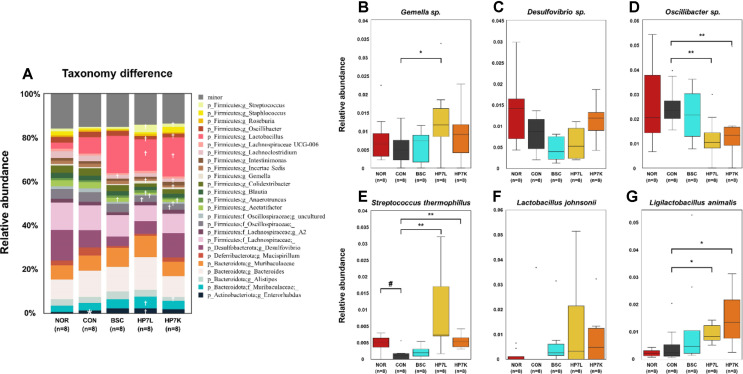
Effects of HP7L and HP7K on fecal microbial taxonomic profiles and selected taxa assigned at the lowest available taxonomic resolution. (**A**) Relative abundance of fecal microbial taxa based on level 6 taxonomic profiles. Taxa assigned to the genus level and taxa assigned to the lowest resolved taxonomic rank are shown. Major taxa with relative abundance ≥ 1% and minor taxa with relative abundance < 1% are indicated. Differential taxa were identified using LEfSe analysis with an LDA score > 3.0. Relative abundances of selected taxa are shown for (**B**) *Gemella* sp., (**C**) *Desulfovibrio* sp., (**D**) *Oscillibacter* sp., (**E**) putative *Streptococcus thermophilus*, (**F**) putative *Lactobacillus johnsonii*, and (**G**) putative *Ligilactobacillus animalis*. Data are presented as box plots. Statistical significance is indicated as *p < 0.05 and **p < 0.01.

Taxonomic changes were further examined using level 6 taxonomic profiles, including genus level taxa and taxa assigned to the lowest resolved taxonomic rank when genus level classification was not available (Fig. 8A). Key microbial biomarkers were identified using LEfSe analysis with an LDA score threshold of > 3.0 (Fig. S1). The CON group showed higher relative abundances of *Enterorhabdus*, *Erysipelotrichaceae*, *Lactococcus*, and *Monoglobus*, compared with the NOR group, along with numerical increases in *Bacteroides* and numerical decreases in *Desulfovibrio*. In the HP7L group, *Lactobacillus*, *Muribaculaceae*, *Streptococcus*, *Lachnospiraceae* NK4A136 group, and *Gemella* were enriched, whereas *Enterococcaceae*, *Butyricicoccus*, *Lactococcus*, *Oscillibacter*, and *Acetatifactor*, were reduced. In the HP7K group, *Lactobacillus* and *Streptococcus* were enriched, while *Monoglobus*, *Lachnoclostridium*, *Alistipes*, *Oscillibacter*, *Lactococcus*, and *Acetatifactor* were reduced.

### Shifts in Selected Taxa Assigned at the Lowest Available Taxonomic Resolution

To further characterize microbial changes, selected taxa assigned at the lowest available taxonomic resolution were analyzed (Fig. 8B–8G). The relative abundance of *Gemella* sp., which decreased in the CON group compared with the NOR group, significantly increased following HP7L administration and showed an increasing trend in the HP7K group, with the highest levels observed in the HP7L group (Fig. 8B). In contrast, *Desulfovibrio* sp. levels were lower in the CON group than in the NOR group and further decreased in the HP7L and BSC groups, whereas HP7K administration restored the relative abundance to a level comparable to that of the NOR group (Fig. 8C). *Oscillibacter* sp. exhibited an increasing trend in the CON group, while its abundance in the BSC group remained similar to that of the NOR group. Notably, both HP7L and HP7K treatments significantly reduced *Oscillibacter* abundance compared with the CON group (Fig. 8D). Similarly, the relative abundance of the taxon putatively assigned as *Streptococcus thermophilus* was significantly reduced in the CON group compared with the NOR group but was markedly restored by both HP7L and HP7K treatments, with the highest abundance observed in the HP7L group (Fig. 8E). The taxon putatively assigned as *Lactobacillus johnsonii* was undetected in the CON group but reappeared in the BSC, HP7L, and HP7K groups, with the greatest recovery observed in the HP7L group (Fig. 8F). Finally, the taxon putatively assigned as *Ligilactobacillus animalis* showed comparable levels among the NOR, CON, and BSC groups but was significantly increased following both HP7L and HP7K administration, with the highest abundance observed in the HP7K group (Fig. 8G).

### Correlation between Gut Microbiota and Physiological Parameters

**Fig. 9:**
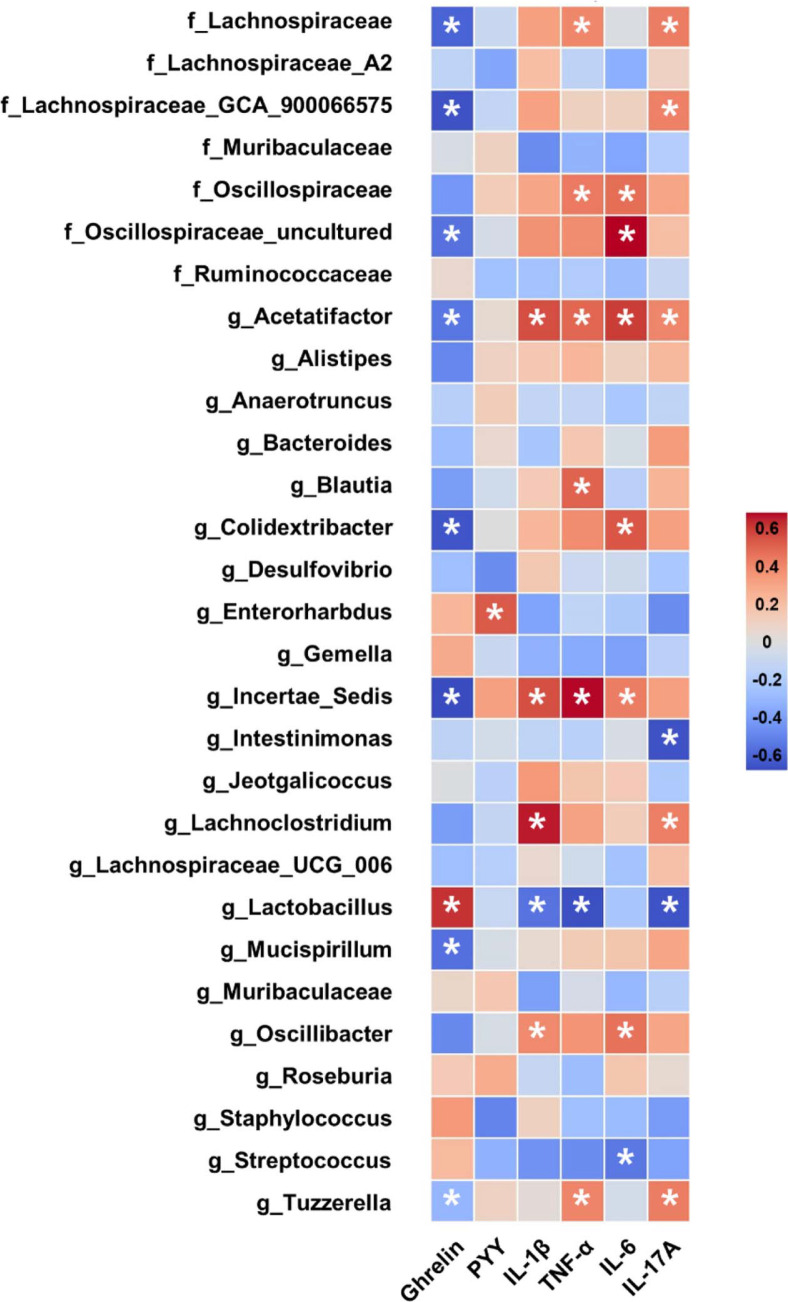
Correlation analysis between gut microbial taxa and host physiological parameters. Spearman’s rank correlation analysis was performed to assess associations between fecal microbial taxa and host parameters, including gastrointestinal hormones and inflammatory cytokines. Positive correlations are shown in red, and negative correlations are shown in blue. Asterisks indicate statistically significant correlations.

Spearman’s correlation analysis was performed to explore associations between gut microbial taxa and physiological parameters, including motility-related hormones and inflammatory cytokines (Fig. 9). Ghrelin showed a significant positive correlation with *Lactobacillus*, whereas significant negative correlations were observed with *Lachnospiraceae*, *Acetatifactor*, *Colidextribacter*, and *Incertae Sedis*. PYY showed a significant positive correlation only with *Enterorhabdus*. For inflammatory cytokines, *Acetatifactor* showed significant positive correlations with IL-1β, TNF-α, IL-6, and IL-17A. In contrast, *Lactobacillus* showed significant negative correlations with IL-1β, TNF-α, and IL-17A. *Lachnoclostridium* was positively correlated with IL-1β and IL-17A, while *Lachnospiraceae* showed positive correlations with TNF-α and IL-17A. *Oscillibacter* was positively correlated with IL-1β and IL-6.

### Predicted Functional Shifts in the Gut Microbiota Following HP7 Administration

**Fig. 10:**
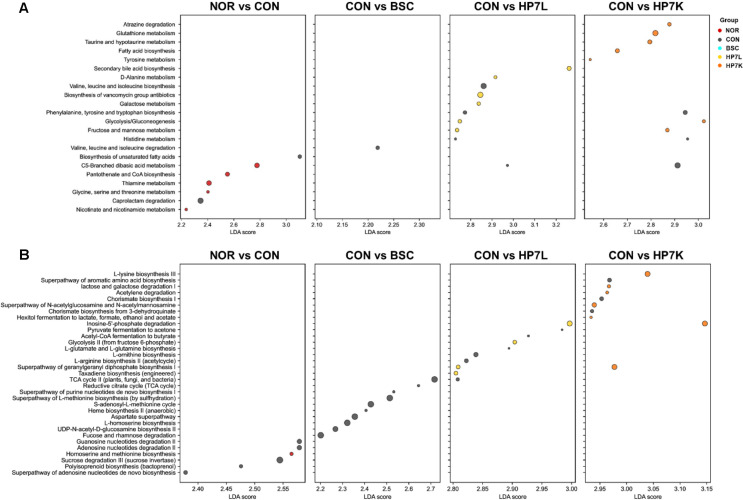
Predicted functional profiles of the gut microbiota following HP7L and HP7K administration. Predicted microbial functional pathways were analyzed using q2-picrust2 and LEfSe. (**A**) KEGG Orthology-based predicted pathway features and (**B**) MetaCyc predicted metabolic pathway features are shown for pairwise comparisons among experimental groups. Differentially enriched predicted features were ranked by linear discriminant analysis (LDA) score, and the top 10 features with the highest LDA scores are presented for each comparison. Colors indicate the experimental group in which each predicted pathway was enriched.

Predicted functional profiles of the gut microbiota were analyzed using *q2-picrust2* and LEfSe based on KEGG Orthology and MetaCyc pathway features (Fig. 10A and 10B).

In the KEGG analysis, the NOR and CON groups showed differences in predicted pathways related to vitamin and cofactor metabolism, amino acid metabolism, and xenobiotic degradation. The CON and BSC groups showed limited differences, with valine, leucine and isoleucine degradation predicted to be enriched in the CON group. Compared with the CON group, HP7L showed predicted enrichment of fructose and mannose metabolism, glycolysis/gluconeogenesis, galactose metabolism, biosynthesis of vancomycin group antibiotics, D-alanine metabolism, and secondary bile acid biosynthesis. HP7K showed predicted enrichment of atrazine degradation, glutathione metabolism, taurine and hypotaurine metabolism, fatty acid biosynthesis, tyrosine metabolism, glycolysis/gluconeogenesis, and fructose and mannose metabolism. In both HP7 comparisons, glycolysis/gluconeogenesis and fructose and mannose metabolism were predicted to be enriched in the HP7-treated groups, whereas phenylalanine, tyrosine and tryptophan biosynthesis was predicted to be enriched in the CON group.

MetaCyc analysis further showed differences in predicted microbial metabolic pathways among groups (Fig. 10B). Compared with the CON group, HP7L showed predicted enrichment of inosine-5'-phosphate degradation, glycolysis II from fructose 6-phosphate, the superpathway of geranylgeranyl diphosphate biosynthesis I, and taxadiene biosynthesis. HP7K showed predicted enrichment of L-lysine biosynthesis III, lactose and galactose degradation I, the superpathway of N-acetylglucosamine and N-acetylmannosamine, hexitol fermentation to lactate, formate, ethanol and acetate, inosine-5'-phosphate degradation, and the superpathway of geranylgeranyl diphosphate biosynthesis II. In both HP7 comparisons, inosine-5'-phosphate degradation and geranylgeranyl diphosphate biosynthesis pathways were predicted to be enriched in the HP7-treated groups.

## Discussion

In the present study, both live and heat-killed *L. paracasei* HP7 improved intestinal motility-related parameters in a loperamide-induced delayed intestinal transit model. Loperamide acts as a peripheral μ-opioid receptor agonist that suppresses enteric smooth muscle contraction, prolongs transit time, and enhances fluid absorption [24, 25]. Consistent with this mechanism, the CON group showed reduced fecal moisture, fecal pellet output, and stool consistency, confirming the induction of impaired intestinal motility. HP7 administration improved several motility-related parameters, with HP7L showing more pronounced effects on fecal moisture content and stool consistency, whereas HP7K significantly improved fecal output and small intestinal transit but showed only numerical improvements in fecal moisture content and stool consistency. These findings are consistent with previous reports that probiotics can improve stool hydration and bowel function through microbial fermentation, intestinal water handling, and motility regulation [26, 27]. The charcoal meal test further showed increased small intestinal transit in the BSC, HP7L, and HP7K groups. Unlike bisacodyl, which directly stimulates enteric nerves and the intestinal mucosa, HP7 may act through gradual modulation of gut microbial and host physiological factors [28, 29]. Serotonin (5-HT), a key regulator of intestinal motility, was reduced by loperamide, whereas HP7L increased serum 5-HT and HP7K showed an increasing tendency. Probiotics have been reported to regulate serotonin production through microbial metabolites, including short-chain fatty acids (SCFAs), and interactions with enterochromaffin cells [30-32]. The response observed in the HP7K group may be related to non-viable microbial components, including microbe-associated molecular patterns (MAMPs), which can interact with epithelial and immune cells and influence host signaling pathways [33]. HP7 also modulated ghrelin and peptide YY (PYY), suggesting that its effects on intestinal transit were accompanied by changes in motility-related hormones [34, 35]. In parallel, HP7 treatment reduced inflammatory cytokine levels and regulated intestinal gene expression associated with neuroendocrine signaling, water transport, and barrier integrity. In the small intestine, HP7 increased *Ghrl* and *Tac1* expression and reduced *Slc6a4* expression compared with the CON group. In the colon, HP7 reduced the loperamide-induced increase in *Aqp3*, a water channel associated with colonic water absorption, and increased *Muc2*, *Tjp1*, *Tjp2*, and *Ocln* expression [36, 37]. Histological analysis further showed that HP7L and HP7K reduced colonic tissue alterations and improved smooth muscle thickness and crypt length. Together, these findings indicate that both live and heat-killed HP7 improved intestinal motility-related parameters, with accompanying changes in neuroendocrine, inflammatory, water transport, barrier-related, and histological markers.

Given the central role of the gut microbiota in intestinal homeostasis, fecal microbiota composition was analyzed. Alpha diversity indices tended to increase following loperamide administration, although the differences were not statistically significant. This pattern may be related to prolonged intestinal transit, as slower transit can increase fecal retention time and microbial richness without necessarily reflecting improved gut status [38]. HP7L significantly reduced Faith’s PD and observed features and produced separation in beta diversity, suggesting a greater effect on overall microbial community structure in the HP7L group. This finding is consistent with the ability of viable probiotics to influence gut ecosystems through microbial interaction, metabolite production, and ecological competition. In contrast, HP7K reduced observed features without marked beta-diversity separation, suggesting a more selective effect on microbial composition. These differences suggest that HP7L and HP7K modulated the gut microbiota through partially distinct patterns, with HP7L showing broader community restructuring and HP7K showing more limited but targeted microbial changes.

Taxonomic analysis further showed that loperamide administration altered several microbial taxa associated with delayed transit and intestinal inflammation. The CON group showed increased abundance of *Erysipelotrichaceae* and *Enterorhabdus*, which have been reported in association with altered intestinal transit and inflammatory conditions [39, 40]. HP7L increased *Lactobacillus*, *Lachnospiraceae* NK4A136 group, and *Streptococcus*, taxa that have been linked to carbohydrate fermentation and SCFA production [41, 42]. HP7L also reduced *Oscillibacter*, *Acetatifactor*, and *Colidextribacter*, which were positively associated with inflammatory markers in the correlation analysis. HP7K showed a partially distinct taxonomic pattern, with enrichment of *Lactobacillus* and *Streptococcus* and reductions in *Monoglobus*, *Lachnoclostridium*, *Alistipes*, *Oscillibacter*, *Lactococcus*, and *Acetatifactor*. These findings suggest that heat-killed HP7 did not simply reproduce the microbial effects of live HP7 but induced selective changes in specific taxa. To further examine these taxonomic changes, species-level analysis was performed, although these findings should be interpreted cautiously because 16S rRNA gene sequencing has limited resolution for species-level assignment. HP7L was associated with restoration of Gemella sp., whereas HP7K increased *Desulfovibrio* sp. toward the level observed in the NOR group. Both HP7L and HP7K reduced *Oscillibacter* sp., a taxon associated with delayed transit-related dysbiosis [45]. In addition, changes in taxa putatively assigned as *Streptococcus thermophilus*, *Lactobacillus johnsonii*, and *Ligilactobacillus animalis* indicated that live and heat-killed HP7 affected different microbial members within the gut ecosystem. Overall, the microbiota results suggest that HP7L exerted a broader effect on microbial community structure, whereas HP7K induced selective taxonomic changes despite the absence of viable cells.

Building on the taxonomic results, correlation analysis provided additional context linking microbial changes with host physiological parameters. Ghrelin was positively associated with *Lactobacillus*, whereas negative correlations were observed with *Colidextribacter* and *Acetatifactor*. These relationships are consistent with previous evidence that lactic acid bacteria can support motility-related hormonal regulation and gastrointestinal function [46]. In contrast, PYY was positively correlated with *Enterorhabdus*, and inflammatory cytokines were positively associated with taxa such as *Acetatifactor*, *Lachnoclostridium*, and *Oscillibacter*. These patterns suggest that specific microbial taxa were associated with altered neuroendocrine and inflammatory status in the delayed transit model. Although causality cannot be inferred from correlation analysis alone, the coordinated changes in microbial composition and host physiological markers after HP7 administration support a close relationship between microbiota modulation and intestinal functional recovery.

Functional prediction using KEGG and MetaCyc was further performed to explore potential metabolic differences associated with HP7L and HP7K. Because these results were inferred from 16S rRNA gene profiles, they should be interpreted as predicted functional changes rather than direct metabolic measurements. In the KEGG analysis, both HP7L and HP7K were associated with enrichment of glycolysis/gluconeogenesis and fructose and mannose metabolism compared with the CON group. These shared features suggest that both live and heat-killed HP7 were linked to increased predicted carbohydrate utilization. In contrast, phenylalanine, tyrosine, and tryptophan biosynthesis was enriched in the CON group in both HP7 comparisons, suggesting that HP7 administration was accompanied by a reduction in predicted aromatic amino acid biosynthesis pathways. Because excessive microbial amino acid fermentation can generate potentially harmful metabolites such as ammonia, phenolic compounds, indole derivatives, and p-cresol, this pattern may reflect a shift away from proteolytic metabolic activity under HP7 treatment [47, 48]. Despite these shared features, HP7L and HP7K showed partially distinct predicted functional profiles. HP7L was associated with enrichment of carbohydrate and energy metabolism-related pathways, including galactose metabolism, glycolysis/gluconeogenesis, and glycolysis II from fructose 6-phosphate. HP7K showed enrichment of pathways related to glutathione metabolism, taurine and hypotaurine metabolism, fatty acid biosynthesis, lactose and galactose degradation, hexitol fermentation, and N-acetylglucosamine and N-acetylmannosamine metabolism. These HP7K-associated pathways are consistent with distinct predicted microbial metabolic features, particularly carbohydrate degradation, lipid-related metabolism, and redox-associated pathways. However, direct metabolite validation is required to confirm these functional changes [49]. Because glutathione metabolism, taurine and hypotaurine metabolism, fatty acid biosynthesis, and carbohydrate degradation pathways have been linked to oxidative balance, epithelial function, and microbial metabolic activity, these predicted pathways may provide a possible functional context for the host responses observed in the HP7K group [50-52]. Both HP7L and HP7K also enriched inosine-5’-phosphate degradation and geranylgeranyl diphosphate biosynthesis pathways in the MetaCyc analysis, indicating partially overlapping functional responses between the live and heat-killed preparations.

The distinct patterns observed between HP7L and HP7K are consistent with their different biological properties. HP7L showed broader changes in microbial community structure, which may reflect the capacity of viable probiotics to interact with resident microbiota through growth, metabolite production, and ecological competition. In contrast, HP7K induced less pronounced changes in beta diversity but improved intestinal transit and selected barrier-related and inflammatory markers, along with changes in predicted microbial functional profiles. This finding supports the possibility that heat-killed HP7 can retain biological activity without viable colonization, potentially through microbial structural components and their interactions with host or resident microbial communities. Therefore, live and heat-killed HP7 may share some functional effects while also acting through partially different microbiota-associated pathways. Several limitations should be considered. Only male BALB/c mice were used in this study, and potential sex-dependent differences in gut motility, microbiota composition, and immune responses should be considered in future studies. In addition, fecal microbiota were analyzed only at the endpoint, and baseline microbiota profiling was not performed. Therefore, individual pre-existing microbial variation could not be fully controlled. Barrier-related markers were assessed only at the mRNA level, and protein-level validation or intestinal permeability assays were not performed. Serum serotonin, ghrelin, and PYY levels provide systemic information, but these measurements do not identify the tissue source or local intestinal signaling pathways. The functional mechanisms proposed in this study were based on 16S rRNA gene sequencing and bioinformatic prediction, and direct quantification of microbial metabolites, including short-chain fatty acids, bile acids, amino acid-derived metabolites, and other luminal metabolites, was not performed. Future studies integrating metabolomics, targeted biochemical analyses, and mechanistic validation are required to confirm the predicted metabolic shifts and clarify causal relationships between microbial function and host physiology. In addition, because the present findings were obtained using a loperamide-induced mouse model, further studies are needed to evaluate the efficacy, optimal dosage, and long-term safety of HP7 preparations in humans.

Overall, this study demonstrated that both live and heat-killed *L. paracasei* HP7 improved delayed intestinal transit and modulated host physiological and microbial parameters. HP7L exerted broader effects on microbial community structure, whereas HP7K showed consistent activity as a heat-killed preparation with distinct predicted functional features. These findings support further evaluation of heat-killed HP7 as a postbiotic candidate for intestinal motility regulation and suggest that viable and non-viable HP7 preparations may have complementary effects on gastrointestinal function.

## Supplemental Materials

Supplementary data for this paper are available on-line only at http://jmb.or.kr.
