# Supplementary material for: Comparative Effects of Live and Heat-Killed Lacticaseibacillus paracasei HP7 on Intestinal Motility, Barrier-Related Markers, and Gut Microbiota in Delayed Transit Mice
Source: J Microbiol Biotechnol. 2026 Jul 1;36:e2605041. doi: 10.4014/jmb.2605.05041 (PMC13364765; doi:10.4014/jmb.2605.05041)
Supplement: Supplementary file 1 [file jmb-36-e2605041-supple.pdf]

J. Microbiol. Biotechnol. 2026. 36: e2605041

https://doi.org/10.4014/jmb.2605.05041

Supplementary Figure

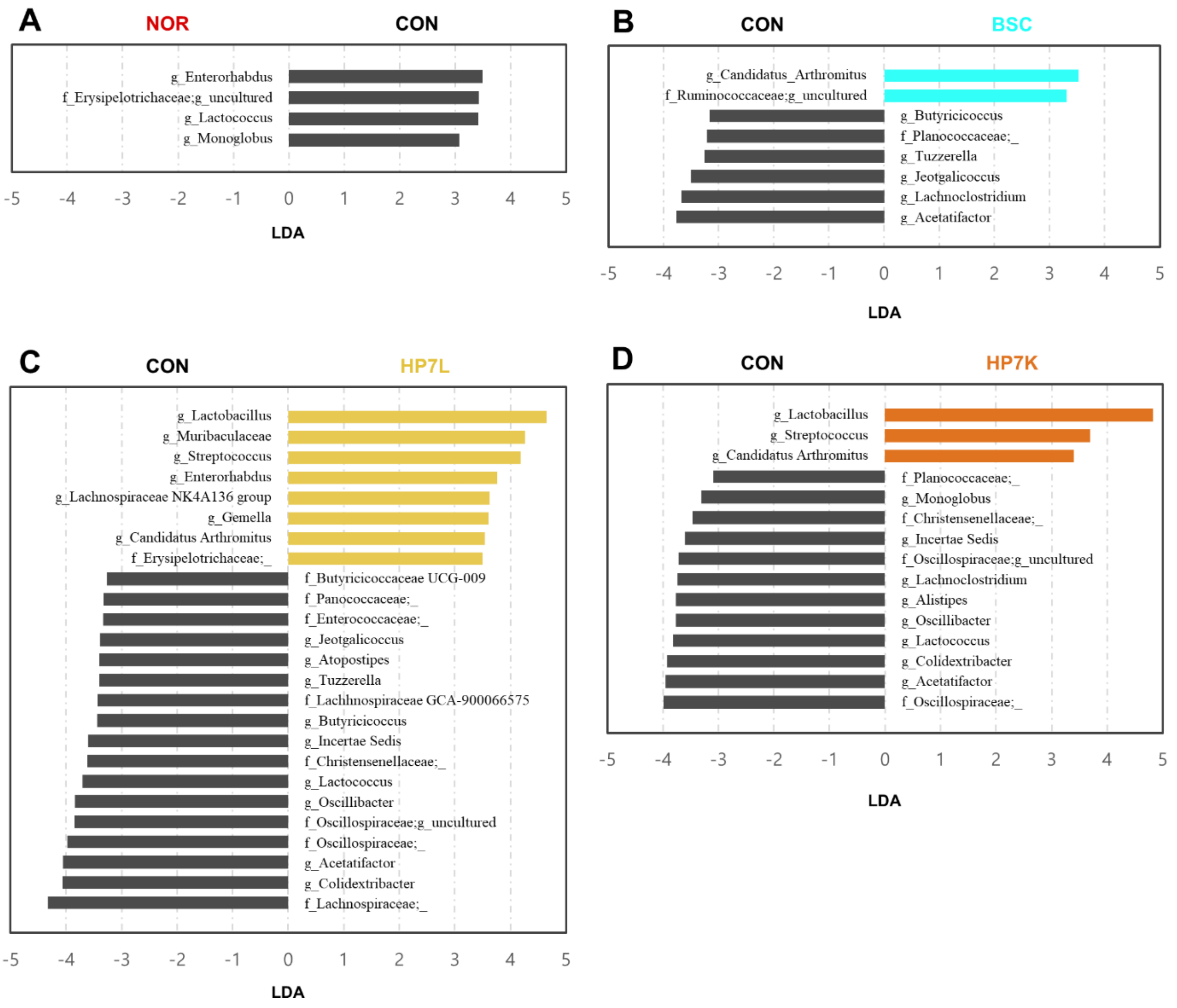

Fig. S1. **LEfSe analysis of fecal microbial taxa among experimental groups. Differentially abundant taxa were identified using LEfSe based on level 6 taxonomic profiles**. Pairwise comparisons are shown for **(A)** NOR versus CON, **(B)** CON versus BSC, **(C)** CON versus HP7L, and (D) CON versus HP7K were compared with the loperamide-induced delayed intestinal transit control group (CON). Taxa with an LDA score > 3.0 are shown.
